# Strength Development and Elemental Distribution of Dolomite/Fly Ash Geopolymer Composite under Elevated Temperature

**DOI:** 10.3390/ma13041015

**Published:** 2020-02-24

**Authors:** Emy Aizat Azimi, Mohd Mustafa Al Bakri Abdullah, Petrica Vizureanu, Mohd Arif Anuar Mohd Salleh, Andrei Victor Sandu, Jitrin Chaiprapa, Sorachon Yoriya, Kamarudin Hussin, Ikmal Hakem Aziz

**Affiliations:** 1 Center of Excellence Geopolymer and Green Technology, School of Materials Engineering, Universiti Malaysia Perlis (UniMAP), P.O. Box 77, D/A Pejabat Pos Besar, 01000 Kangar, Perlis, Malaysia; arifanuar@unimap.edu.my (M.A.A.M.S.); sav@tuiasi.ro (A.V.S.); kamarudin@unimap.edu.my (K.H.); ikmalhakem@gmail.com (I.H.A.); 2 Faculty of Materials Science and Engineering, “Gheorghe Asachi” Technical University, Blvd. D. Mangeron 71, 700050 lasi, Romania; 3 Romanian Inventors Forum, Str. Sf. P. Movila 3, Iasi 700089, Romania; 4 National Institute for Research and Development in Environmental Protection, 294 Splaiul Independenței Blv, 060031 Bucharest, Romania; 5 Synchrotron Light Research Institute (SLRI), 111 University Avenue, Muang District, Nakhon Ratchasima 30000, Thailand; jitrin@slri.or.th; 6 National Metal and Materials Technology Center (MTEC), 114 Thailand Science Park, Phaholyothin Road, Klong 1, Klongluang, Pathumthani 12120, Thailand; sorachy@mtec.or.th

**Keywords:** dolomite/fly ash, geopolymer, strength development, temperature exposure, Micro-XRF

## Abstract

A geopolymer has been reckoned as a rising technology with huge potential for application across the globe. Dolomite refers to a material that can be used raw in producing geopolymers. Nevertheless, dolomite has slow strength development due to its low reactivity as a geopolymer. In this study, dolomite/fly ash (DFA) geopolymer composites were produced with dolomite, fly ash, sodium hydroxide, and liquid sodium silicate. A compression test was carried out on DFA geopolymers to determine the strength of the composite, while a synchrotron Micro-Xray Fluorescence (Micro-XRF) test was performed to assess the elemental distribution in the geopolymer composite. The temperature applied in this study generated promising properties of DFA geopolymers, especially in strength, which displayed increments up to 74.48 MPa as the optimum value. Heat seemed to enhance the strength development of DFA geopolymer composites. The elemental distribution analysis revealed exceptional outcomes for the composites, particularly exposure up to 400 °C, which signified the homogeneity of the DFA composites. Temperatures exceeding 400 °C accelerated the strength development, thus increasing the strength of the DFA composites. This appears to be unique because the strength of ordinary Portland Cement (OPC) and other geopolymers composed of other raw materials is typically either maintained or decreases due to increased heat.

## 1. Introduction

Geopolymers refer to binder materials that are generated through the activation of aluminosilicate materials with alkali or alkali–silicate solutions [1,2]. Geopolymers that are otherwise inorganic polymers or alkali-activated binders have garnered interest at the global level [3]. Generally, geopolymers are amorphous to semi-crystalline and three-dimensional silica alumina-based materials. Geopolymers can be produced by mixing aluminosilicate precursor materials, such as fly ash, kaolin or metakaolin, metal slag, and dolomite, with strong alkali solutions. The common alkali solutions used to produce geopolymers are sodium hydroxide (NaOH), potassium hydroxide (KOH), sodium silicate, and potassium silicate [4]. The geopolymer paste can be cured either at room temperature or an elevated temperature. The aluminosilicate source dissolves and forms free Si^4+^, Al^3+^ tetrahedral units and Ca^2+^, under strong alkali solution. The progress of the reaction continues with the slow removal of water. Next, SiO_4_, AlO_4_ tetrahedral, and CaO clusters are integrated to produce polymeric precursors through sharing all oxygen atoms. This will eventually form amorphous or semi-amorphous geopolymers. The primary block of the geopolymer chain is Si-O-Al [5]. Basically, geopolymers are a man-made material that offer several advantages, including good mechanical strength (similar or more than ordinary Portland Cement (OPC)) and the capacity to encapsulate hazardous waste, as well as being water- and fire-resistant [6]. The strength of geopolymers is a critical factor within the building and construction domain. The development of strength in geopolymers strongly depends on the raw materials and the alkali activator solutions [7,8,9,10,11,12,13,14,15,16]. Even though geopolymers with slow strength development generate materials with lower strength, this drawback can be addressed by increasing heat and aging time [17]. 

The compressive strength of materials after being exposed to elevated temperatures appears to be one of the aspects of concern for application in the building and construction domain. A prior study assessed the strength of geopolymers after they were exposed to elevated temperatures using Class F fly ash [18]. The strength of geopolymer composites reduced after the temperature was increased from 100 to 800 °C. The strength went far lower than the strength of the geopolymer that was not exposed to an elevated temperature. Another study on exposure to elevated temperature was performed by comparing the strength of geopolymer (fly ash) with OPC composites [19]. As a result, the compressive strength of both materials deteriorated gradually. It is noteworthy to highlight that the OPC composites displayed the worst decline in strength, when compared to that of the geopolymer composite. Sodium hydroxide (NaOH) concentration (part of the alkali solution) greatly affected the dissolution process of silica and alumina from fly ash, wherein increased molarity led to an increased dissociation of the active species of raw material, apart from yielding the formation of more geopolymer gel network [20]. Another crucial factor in generating geopolymers refers to the ratio of NaOH to sodium silicate (Na_2_SiO_3_). Morsy et al., reported that increments in compressive strength were affected by the NaOH/Na_2_SiO_3_ ratio, which escalated from 1.0 to 2.5 [21].

In a previous study, dolomite was only used as a filler and replacement for other raw materials in alkali-activated composites and geopolymer composites. However, no study has applied dolomite as the major or main raw material in geopolymer composites. Zarina et al., [22] used dolomite only as an addition to boiler ash, which turned into geopolymer paste, while Yip et al., [23] applied dolomite merely as a carbonate mineral addition to a metakaolin-based geopolymer. Dolomite has the potential to be used as geopolymer raw material due to its Al, Si, and Ca contents. Nonetheless, the incorporation of dolomite in geopolymer is still new and in its infancy within the research domain. Information concerning dolomite geopolymer is in scarce, especially in terms of strength development under exposure to elevated temperature. Therefore, this study investigated the effect of elevated temperature exposure on geopolymer composites based on dolomite/fly ash (DFA). The study outcomes enhance comprehension regarding the mechanical properties for the future improvement and application of dolomite geopolymers, especially those exposed to elevated temperatures.

## 2. Experimental

### 2.1. Materials

Dolomite was applied as the geopolymer raw material in this study. The dolomite was supplied by Perlis Dolomite Industries Sdn. Bhd., Perlis, Malaysia. The chemical composition of dolomite was tabulated in Table 1. The size of the dolomite was set to below 63 μm. The hardness of the dolomite ranged from 3.5 to 4.0 (Mohs hardness) with a specific gravity between 2.8 and 2.9. The solid dolomite was ground to obtain its powder form with irregular particle shapes. The resultant dolomite was used as a raw material in the investigation carried out in this study.

F class fly ash was gathered from a coal combustion plant located in Manjung, Perak, Malaysia. Fly ash refers to waste generated from YTL Corporation Berhad. The size of fly ash was fixed at below 63 μm. The collected fly ash was in a fine powder form with a generally spherical shape. The fly ash was applied as a raw material for this study purpose. The chemical composition of fly ash was tabulated in Table 2. 

The NaOH used in this study refers to caustic soda flakes called Formosoda-P supplied by Formosa Plastic Corporation, Taiwan. The NaOH in flake form was diluted in water to form alkaline solution. The molecular weight of the NaOH was 40 g/mol with 99.0% purity. The use of flake-type sodium hydroxide gave a solution with high purity (can be controlled during diluting process), when compared to that in liquid form. 

The technical grade of liquid Na_2_SiO_3_ was supplied by South Pacific Chemical Industries Sdn. Bhd. (SPCI), Malaysia. The liquid Na_2_SiO_3_ is colorless and dissolves readily in 60.5% water. Sodium silicate, which is readily soluble in water, appears to be the most silicon-rich when compared to its powder form. The Na_2_SiO_3_ is composed of 30.1% of silica and 9.4% of sodium oxide. The molecular weight of Na_2_SiO_3_ is 122.06 g/mol. Both the silica content and the viscosity of this sodium silicate are suitable for application in geopolymer. Sodium silicate was applied as part of the alkali activator solution in this study.

### 2.2. Sample Preparation

NaOH solution with 22M concentration was prepared in a volumetric flask, which was put in the circulate water bath to ensure the cooling down of the solution. The NaOH solution was mixed with Na_2_SiO_3_ solution with a Na_2_SiO_3_/NaOH ratio of 2.5 to formulate the alkali activator solution 24 hours prior to analysis. Next, DFA with a 60/40 ratio and an alkali activator solution was mixed with a solid to liquid ratio of 2.5 and stirred well by using a mechanical mixer. The fresh paste was rapidly poured into steel mold and was compressed into each cube compartment at each layer by adhering to ASTM C109. The samples were oven-dried for 24 hours at 80 °C for curing purposes. During the curing process, the samples were sealed with thin plastic at the exposed part of the mold. The samples were exposed to elevated temperatures from 200 to 1000 °C after 28 days of curing, in order to achieve the study objective.

### 2.3. Testing and Chracterization Method 

Instron machine series 5569 Mechanical Tester was employed to assess the compressive strength of all specimens. The specimens referred to geopolymer composites that were taken out from the oven after 24 hours of curing and were placed at room temperature until the day of testing for control sample, wherein the samples were already exposed to elevated temperature (200–1000 °C). The compressive test was performed to examine the strength development of the specimens. The samples were tested after seven days of curing, in which three specimens were tested for each parameter. 

Microstructural characterization of DFA geopolymer composites was performed using the JSM-6460LA model Scanning Electron Microscope (JEOL) with secondary electron detectors. In microstructural analysis, both dolomite and fly ash powder was sprinkled onto double-sided carbon taped prior to analysis, whereby the blower was used to discard loosely held powder. In microstructural analysis, the samples were taken from the surface of the internal structure of the geopolymer breakage prior to compressive test. The samples was were prepared with a size of up to 50 mm × 50 mm × 10 mm and coated with palladium. The coated samples were placed in SEM chamber for characterization process. 

A Micro X-Ray Fluorescence (XRF) machine using a source from synchrotron radiation at beam line 6b (BL6b) of the Synchrotron Light Research Institute (SLRI) was applied to determine the chemical composition and the elemental distribution of the reaction products. The BL6b exploited the continuous synchrotron radiation that emitted from the bending magnet. The specimen was positioned on a motorized stage with three-degree freedom. This Micro-XRF employed an 8 keV radiation from synchrotron, along with three gate valves. The Micro-XRF station used a capillary half-lens for X-ray focusing. This optic focused on X-ray beam from 5 × 2 mm (H × V) at the entrance of the lens down to a diameter of 50 µm measured at a sample positioned 22 mm (lens focal point) downstream of the lens exit. AMPTEK single-element Si (PIN) solid-state was installed to detect the fluorescence element. 

Perkin Elmer FTIR Spectrum RX1 Spectrometer was used to determine the functional groups of a DFA geopolymer composite. The samples were analyzed using the attenuated total reflectance (ATR) technique. The samples were crushed into powder form using a mortar. Initially, the specimens were placed on the sample slot (ATR crystal) located at the sample platform of the machine. The pressure tower that contained the compression tip was moved to the top of the specimens and closed tightly. The specimens were scanned between 500 and 4000 cm^−1^ with a resolution of 4 cm^−1^. The sample spectrum was collected after gathering the background spectrum.

An XRD-6000 Shimadzu X-Ray diffractometer was applied to characterize the DFA geopolymer composite. The x-ray diffractometer (XRD) was equipped with auto-search/match software as standard to identify the crystalline phases. The specimens were in powder form. The samples were prepared in a size that ranged from 1 to 63 µm. Extremely fine grain specimens were required for the analysis of powders via XRD to attain good signal to noise ratio, avoid fluctuations in intensity and spottiness, and to reduce the preferred orientation. The XRD analysis was performed using Cu-Kα radiation with X-ray tube that operated at 40 kV and 35 mA. The XRD data were collected at 2θ values in the range of 10° to 90° at a scan rate of 2° per minute and scan steps of 0.02°(2θ). Good phase identification may be made if at least three of the main diffraction peaks of the unknown phase match the standard diffraction pattern of a known crystal phase retrieved from the literature of powder diffraction database.

## 3. Results and Discussions

### 3.1. Microstructure Analysis

**Figure 1:**
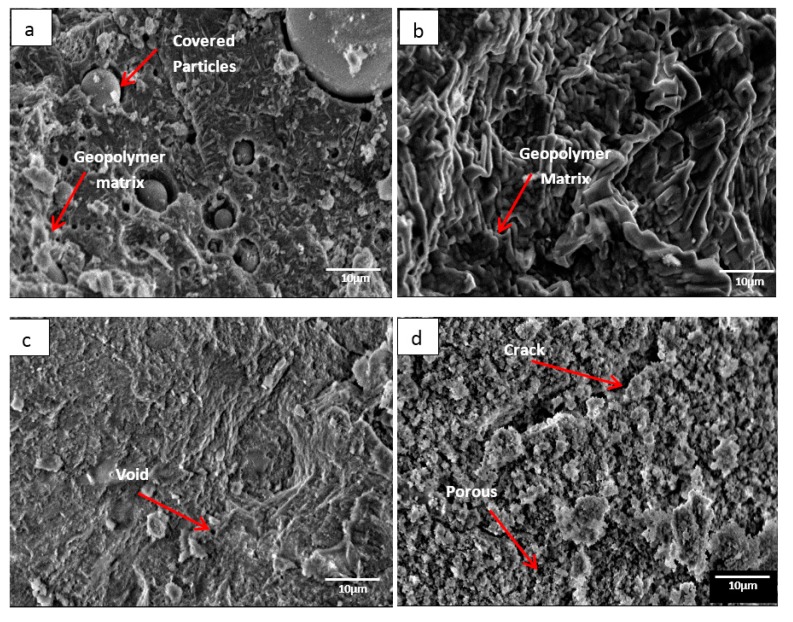
Microstructure of dolomite/fly ash geopolymer composite (**a**) before temperature exposure, after fire exposure at (**b**) 200, (**c**) 400, and (**d**) 1000 °C.

Figure 1 illustrates the microstructure of DFA geopolymer composites for (a) before temperature exposure, as well as after temperature exposure, at (b) 200, (c) 400, and (d) 1000 °C. In Figure 1a, the microstructure signifies that the geopolymer matrix was not fully developed in the system, mainly because some raw materials retained their original shape (e.g., spherical fly ash). The microstructure in Figure 1b displays no occurrence of crack on the surface of the geopolymer composite. The growth of geopolymer matrices began to continue when heat was supplied from the exposure. Based on Figure 1c, the microstructure exhibits the occurrence of small voids. The geopolymer that served as a binder in the DFA composite was completely cured. In Figure 1d, cracks are noted on the structure of the DFA composite, which also signifies porosity. The microstructure in Figure 1 exemplifies the development of the geopolymer matrix and the increased density of the geopolymer structure from (a) to (c), whereas porosity and density decreased from (c) to (d). 

In Figure 1b, the growth of the geopolymer matrix began to take place as heat was continuously supplied. Dolomite has a high amount of calcium (Ca) content. During the dissolution process of geopolymer, the CaO content in dolomite was attacked by the active species from alkali solution and turned into active species of Ca^2+^ and O^2−^. The Ca^2+^ ions, along with Si^4+^ and Al^3+^ that reacted to OH^−^ ions from alkali solution, formed a geopolymer composite. At this stage, the Ca content in dolomite began promoting the optimum reaction within the geopolymer system. Increments in temperature accelerated the continuous development of geopolymer matrices, thus increasing the strength of DFA composites. 

As the heat increased due to the increasing temperature, the geopolymer matrix in Figure 1c accelerated in its growth. The structure turned out to be dry and smooth, along with some void due to water evaporation. As the polycondensation reaction continued, a gel network underwent the rearrangement process in geopolymerisation. This promoted the geopolymer matrices to continue in its growth and to fill the space within the geopolymer composite structure (space-filling gel) [24]. 

In Figure 1d, a crack is noted and the structures of the geopolymer composite turned porous. This was due to the increase in heat supply to the sample. At this point, shrinkage started to take place due to dehydration and dehydroxylation. The dehydroxylation process included the heating process, through which the hydroxyl group (OH) was released by forming a water molecule. Distortion and buckling of the polymeric structure of aluminosilicates appeared to be the consequence of the release of structural water via dihydroxylation, which resulted in a disordered structure [25]. Equation (1) shows the general mechanism of dehydroxylation in geopolymers.

OH^−^ ↔ H^+^ + O^2−^ H^+^ + OH^−^ ↔ H_2_O (1)

The high stresses in the void wall developed the movement of non-combined water, thus the greater degradation occurred with rising temperature due to fine pores and shrinkage [26]. This was also due to breakage of the bonding and phase changing from an amorphous to a crystalline phase. 

### 3.2. Elemental Distribution Analysis 

**Figure 2:**
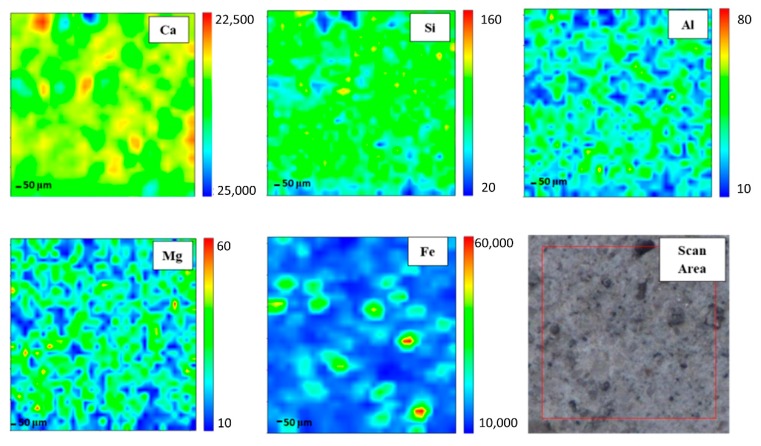
Micro-XRF elemental mapping of Ca, Si, Al, Mg and Fe in a ~961 × 50 μm overview of dolomite/fly ash geopolymer composites unexposed at elevated temperature.

**Figure 3:**
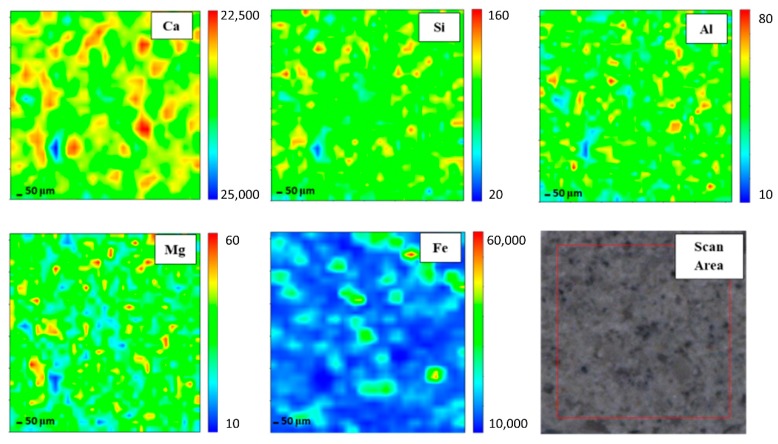
Micro-XRF elemental mapping of Ca, Si, Al, Mg and Fe in a ~961 × 50 μm overview of dolomite/fly ash geopolymer composites after exposure at 400 °C.

In order to gain elemental maps, Micro-XRF that used a synchrotron source was applied. In this investigation, the Ca, Si, Al, Mg, and Fe maps were determined. Micro-XRF maps of a ~961 × 50 μm overview of DFA geopolymer composites before (control) and after exposure to 400 °C are presented in Figure 2 and Figure 3, respectively. The sample exposed to 400 °C was selected as it recorded the highest strength amongst the rest. Generally, all the elements in DFA geopolymer composites are well distributed. Some variances were noted in the maps of elemental distribution between composites that were not exposed and those exposed to 400 °C. Based on Figure 3, the elemental distribution of composite that was exposed to 400 °C displayed an increment in the amount of distribution region, when compared to the composite unexposed to elevated temperature. The elements of Ca, Si, and Al in the maps portrayed some increment in the red region (high concentration) after being exposed to 400 °C. The combination of Si and Al maps showcased the formation of Si-O-Al, which emerged as one of the most important bonds that determined the strength of the geopolymer. The green and yellow regions (medium concentration), along with a high-concentration region for Si and Al elements, had more spread in the map of the composite that was exposed to 400 °C. The magnesium (Mg) element also increased after exposure to 400 °C, while the Fe element displayed slight changes in the concentration region. The blue region (low concentration) indicates no element or void in the composite. The green and yellow regions (medium concentration) signify a reaction between elements within the composite. The high-concentration region or the red region reflects a reaction between the investigated elements that leads to the formation of a phase.

A high amount of Ca in geopolymer usually produces a good setting time. Despite material with high Ca content obtaining a better setting time compared to other geopolymers with low Ca content, the strength development was found to be slow. Strength is gained with an increase in curing time and curing temperature. The occurrence of Si and Al elements in geopolymer composites affects the strength development. Increasing Si and Al elements increases the strength development of the geopolymer composites as more geopolymer chains are created. The geopolymer’s main structure consists of Si-O-Al, which clearly shows the important of Si and Al elements in producing good strength development. However, the occurrence of Mg in the geopolymer retarded the strength development of the geopolymer. The occurrence of this has disturbed the backbone structure of Ca-Si-O-Al, thus disturbing the strength development of the geopolymer. With enough Ca, the increased bulk of Mg promotes the formation of low Al C-(A)-S-H due to the formation of hydrotalcite group phases and a reduction in the available Al element. Hydrotalcite group phase formation is linked to the increase in C-(N)-A-S-H gel polymerization, decreased gel Al uptake and increased formation of the third aluminate hydrate. MgO or Mg(OH) content affects the properties of geopolymer [27,28]. 

Well-distributed elements in the geopolymer composite samples indicated the production of a homogenous geopolymer composite. A homogenous mixture of geopolymer composites enhances the strength of the material. The combination of Ca, Si, and Al maps led to the formation of a calcium aluminate hydrate (C-A-S-H) phase [29]. An increased high concentration region for Ca, Si, and Al in the map of the composite after exposure to 400 °C reflects an increment in the C-A-S-H phase in the composites. Geopolymer composites that contained Ca element generated the C-A-S-H phase after they went through the geopolymerisation process. Heat supply enhanced the strength development of geopolymer composites. With increased strength development, the formation of the C-A-S-H phase increased. This is proven by the formation of more geopolymer matrices in the microstructure of samples exposed to 400 °C.

The increasing green and yellow regions (medium concentration), along with the high-concentration region for Si and Al elements of the composite exposed to 400 °C, signified the increase in the Si-O-Al bond in geopolymer. Upon increments in the exposed temperature, more geopolymer chains were formed, offering more strength to the composites. The geopolymer consists of repeating units of Si-O-Al. The formation of more Si-O-Al bonds confers more strength to the geopolymer.

The element of Mg increased after the increment in exposed temperature. The occurrence of Mg led to the formation of a hydrotalcite group that may weaken geopolymer strength. Nevertheless, the strength of the composite continued to increase due to the increment in Si and Al elements. At this stage, the Mg element began to grow within the composites. This Mg reacted with other element(s) that occurred from the breakage of the geopolymer structure due to an increase in temperature from 400 to 1000 °C. Due to the increasing pressure in the geopolymer sample as the exposed temperature was increased, both dehydration and dehydroxylation processes took place. Dehydroxylation in clay-occurred at temperatures that ranged between 500 and 700 °C [30]. This led to the breakage of several geopolymer bonds, hence the occurrence of more Si and Al elements [31]. These free elements reacted with Mg and formed new crystalline phases that comprised Mg, Si, and Al, which is further discussed in later phase analysis. The new crystalline phases, such as Akermanite, Juanite, Clinohumite and Epsomite, which consisted of Mg, occurred after the dehydroxylation process in the geopolymer composite. Some crystalline phases weakened the DFA geopolymer composite’s strength. The Fe element exhibited slight changes in the concentration region, mainly because only a small amount of Fe was produced from the raw material (fly ash) [32]. 

### 3.3. Phase Analysis 

**Figure 4:**
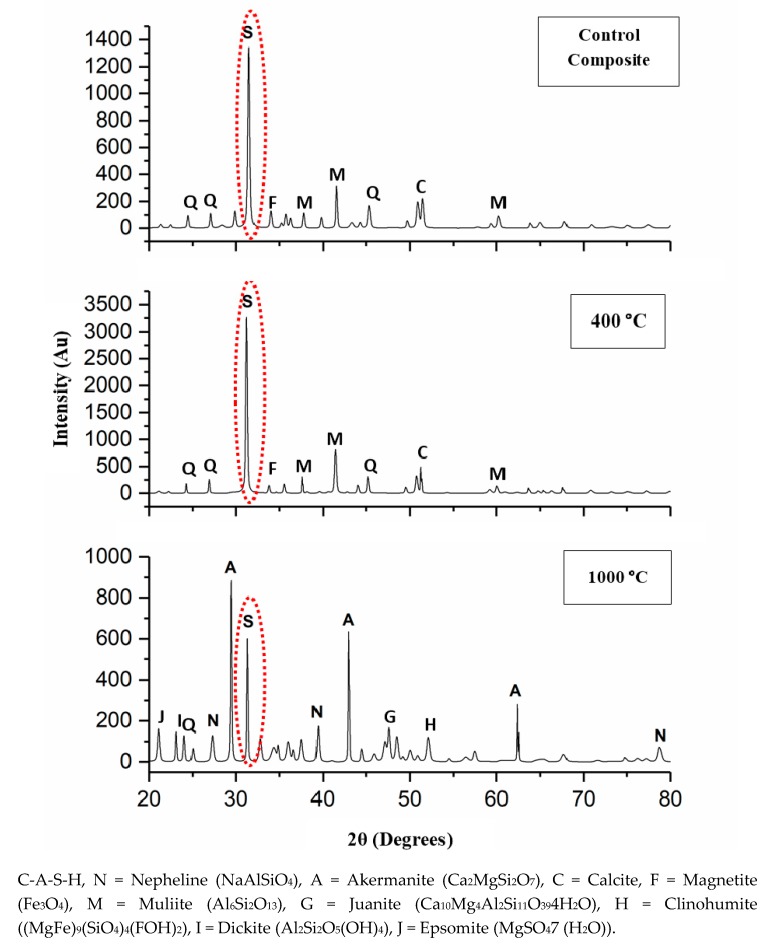
X-Ray Diffraction (XRD) pattern of dolomite/fly ash geopolymer before (Control) and after exposure at 400 and 1000 °C.

Figure 4 illustrates the XRD pattern of the DFA geopolymer composite before (Control) and after being exposed to 400 and 1000 °C. By referring to Figure 4, y axis was indicated to the various intensity obtained while x-axis represented to diffraction angle of mineral phase appearance. The original sample of the XRD patterns portrayed the semi-crystalline phase of the DFA geopolymer composite. The crystalline C-A-S-H peak (ICDD 01-081-1448) was detected in the original geopolymer composite, while the binders of semi-crystalline, Na-geopolymer, Ca-geopolymer, and C-A-S-H gel were discovered in the DFA geopolymer composite system. The control composites were composed of calcite (ICDD 00-003-0670), quartz (ICDD 01-079-1906), mullite (ICDD 01-079-1906), and magnetite (ICDD 01-089-0950). 

As the DFA composite was heated to 400 °C, the intensity of the C-A-S-H peak (2θ = 32°) was increased. This was due to the growth of the C-A-S-H gel, along with the removal of free water. Upon increments in exposure temperature, the C-A-S-H gel continued to grow and develop. The increased exposure temperature accelerated the strength development of the composites. This is attributable to the microstructure of composites exposed to 400 °C, which displayed a greater development of geopolymer matrices that was fully cured. At this point, the phase of the geopolymer remains the same, except for the intensity of the C-A-S-H peak. The formation of a new crystalline reaction phase that still did not occur signified the structural evolution of the C-A-S-H gel, hence the increased composite strength that resulted later. Calcite was formed due to the excessive amount of CaO that reacted with CO_2_, while the magnetite phase took place due to the small amount of Fe contained in the chemical composition of fly ash [33]. The presence of the hydrosodalite phase indicated some geopolymerisation, but with low strength [34].

After the DFA, the geopolymer composite was exposed up to 1000 °C, and the formation of a new crystalline product or peak was clearly noted. Both akermanite (Ca_2_MgSi_2_O_7_) (ICDD 01-074-0990) and nepheline (NaAlSiO_4_) (ICDD 01-076-1858) phases were predominantly formed. Akermanite (Ca_2_MgSi_2_O_7_) refers to a melilite mineral of the sorosilicate group that consists of calcium, magnesium, silicon, and oxygen. Akermanite is a product of contact metamorphism (a change in mineral in existing rocks that occurs preliminarily due to heat and pressure) of siliceous limestones and dolostones (dolomite). The crystalline phases of clinohumite ((MgFe)_9_(SiO_4_)_4_(FOH)_2_) and epsomite (MgSO_4_(H_2_O)) also occurred. Clinohumite is an uncommon member of the humite group that contains magnesium silicate. Its empirical formula reflects four olivines (Mg_2_SiO_4_) and a brucite (Mg(OH)_2_). Clinohumite has brittle properties that normally lead to weak strength if occuring in the composite. Epsomite was originally a hydrous magnesium sulphate that is classified in the orthorhombic system of crystallization and normally forms as efflorescence. Epsomite absorbs water from the air and converts it to hexahydrate with the loss of a water molecule and a switch to a monoclinic structure (unequal length system). Both of these crystalline phases that occurred after the exposure of the composite to 1000 °C contained Mg element.

**Figure 5:**
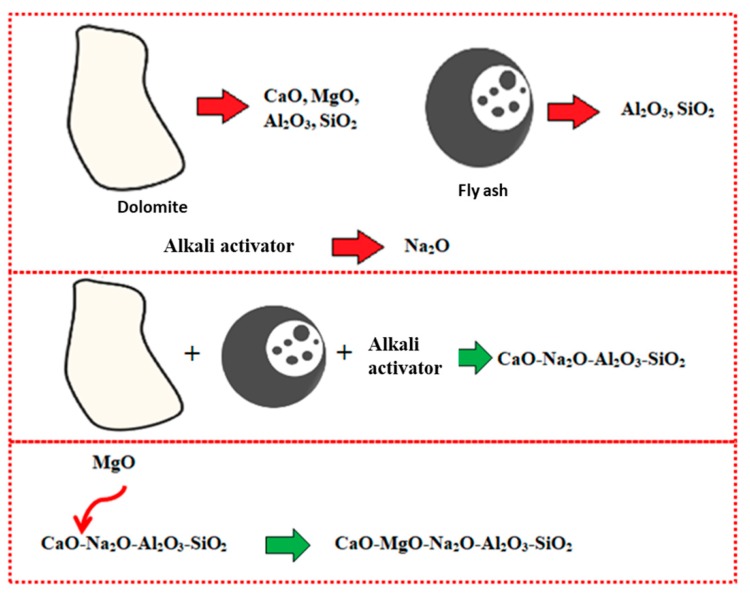
Schematic of Magnesium Oxide (MgO) interference in geopolymer system.

The occurrence of Mg, along with the high composition of Ca, disrupted the geopolymer chain structure and decreased its strength [35,36]. Figure 5 illustrates the interference of Mg content in the geopolymer system. Crystalline phases of nepheline are commonly detected from XRD diffractograms of geopolymer samples after being exposed to elevated temperature. Nepheline is known as a thermally stable material. The presence of crystalline phases that are thermally stable is critical for the thermal stability of the geopolymer structure. Nevertheless, the presence of nepheline in the DFA geopolymer composite was minimal and had low intensity, when compared to the akermanite phase.

The intensity of the C-A-S-H peak decreased due to the decomposition of some geopolymer phases via dehydroxylation process. The dehydration and dehydroxylation caused shrinkage to DFA geopolymer composites. This is closely linked with the occurrence of the crack noted in the composite exposed to 1000 °C in the microstructure analysis result presented earlier. The peak of nepheline (NaAlSiO_4_) was observed as the geopolymer composite was exposed to from 900 to 1000 °C [37]. The peak of dickite (Al_2_Si_2_O_5_(OH)_4_) was also formed from the breakage structure of the DFA geopolymer composite. As the temperature was elevated up to 1000 °C, some new crystalline phases were simultaneously produced, such as akermanite in geopolymer-based metakaolin/GGBS [38]. 

### 3.4. Functional Group Analysis 

**Figure 6:**
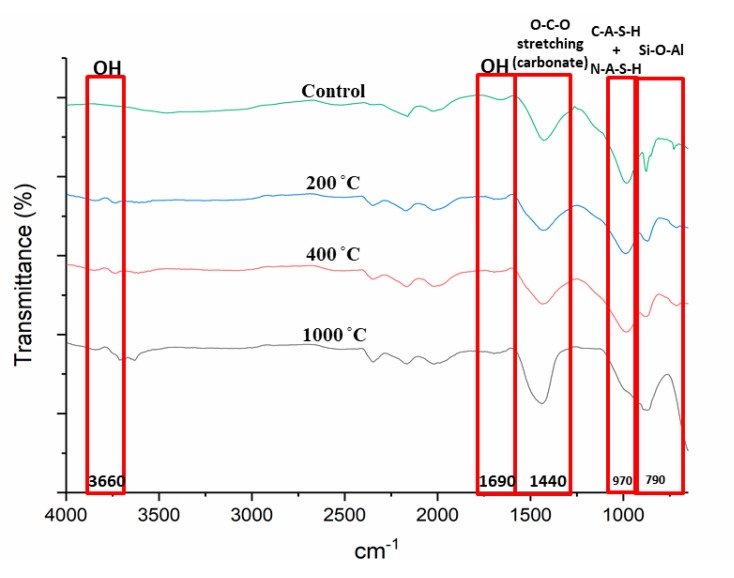
FTIR spectra of dolomite/fly ash geopolymer before (control) and after exposure to 200, 400, 1000 °C.

Figure 6 portrays the Fourier Transform Infrared (FTIR) spectra of DFA geopolymer composites before (control) and after exposure to 200, 400, and 1000 °C. Based on the spectrum displayed in Figure 6, peak changes occurred at bands 3600 and 1690 cm^−1^ for all samples exposed to a high temperature. The peak at 3600 cm^−1^ became broader due to high transmittance and, at 1690 cm^−1^, the peak turned almost flat. The peak at 1400 cm^−1^ increased and shifted to 1450 cm^−1^ as the temperature increased to 1000 °C. Peaks at 970 and 790 cm^−1^ also experienced changes in the control DFA composite as the temperature increased. A change was noted at band 450 cm^−1^ after the composite was exposed to an elevated temperature. 

Changes in peaks occurred at bands 3600 and 1690 cm^−1^ for all samples exposed to a high temperature, mainly due to the removal of free water in the DFA. Bands ranging from 3550 to 3700 and 1690 cm^−1^ corresponded to OH^-^ stretching vibrations, which represented the water molecules present in the material [39]. 

A shift in the peak from 1400 to 1450 cm^−1^ was observed as the temperature was increased to 1000 °C. As the peak shifted to 1450 cm^−1^, the strength of the composite decreased. The band at 1450 cm^−1^ reflects the characteristic of the asymmetric O-C-O stretching mode, which suggests the presence of sodium carbonate as a result of the reaction between excessive sodium and atmospheric carbon dioxide. This formation was due to the atmospheric carbonation on the surface of the matrix, as it reacted with CO_2_ due to the exposure to heat. Zaharaki et al., [40] and Assaedi et al., [41] also reported the occurrence of a peak at 1440 cm^−1^ in the spectra of the samples subjected to heat, which is attributed to the presence of atmospheric carbonation.

The change in peaks at 970 and 790 cm^−1^ from the original DFA composite due to an increase in temperature was clearly observed. As the temperature was elevated, some geopolymer bonds were broken and resulted in the reduction in composite strength. This explains the low compressive strength of the geopolymer composite under 1000 °C heat, which is discussed later. This is also associated with the decreased intensity of the C-A-S-H peak due to the decomposition of some geopolymer phases in the phase analysis result. A band at 970 cm^−1^ was attributed to the formation of C-A-S-H with N-A-S-H gel, while the 790 cm^−1^ bands were assigned to Si–O–Si symmetrical stretching. The magnitude of these bands is attributable to the amorphous nature of the material. The Si-O-Al stretching vibration band is also located at 970 cm^−1^. The Si-O-Al was determined by the peaks found between 700 and 1000 cm^−1^ [42]. 

The band at 450 cm^−1^ experienced a change after the composite was exposed to an elevated temperature. This band is related to Al-O or Si-O in bending and plane mode. The clear change was noted at composites exposed to 1000 °C, which led to the breakage of the Si-O-Al bond that later turned into either Si-O or Al-O.

### 3.5. Compressive Strength 

**Figure 7:**
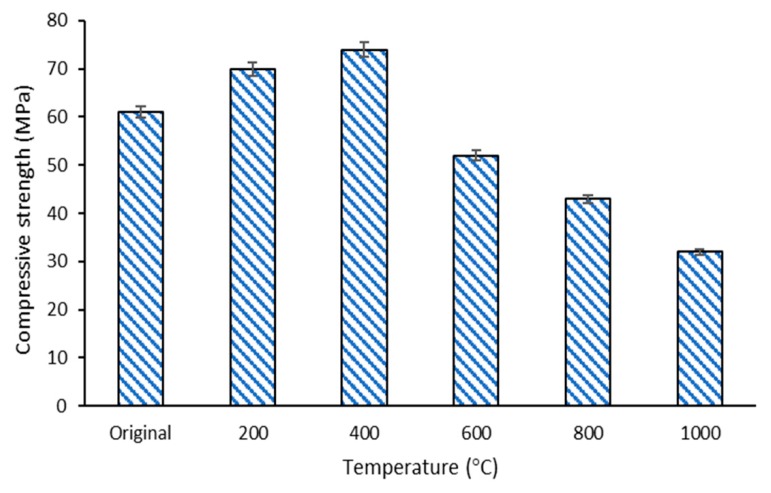
Compressive strength of dolomite/fly ash geopolymer composites before and after fire exposure at 200, 400, 600, 800, 1000 °C.

The result of the compressive strength noted in the composite before (control) and after the temperature exposure at 200, 400, 600, 800, and 1000 °C is shown in Figure 7. The graph displays that the compressive strength of DFA composites increased as the exposed temperature increased to 400 °C. At 400 °C, the compressive strength of the DFA geopolymer composites was 23% higher than that of the original (without temperature exposure) DFA composites. However, the strength of the composites began to decrease when the exposure temperature was elevated from 600 to 1000 °C. The DFA composite at 1000 °C exhibited lower compressive strength than those recorded at other temperatures. The highest strength was 74.48 MPa, after exposure to 400 °C, while the lowest strength was 32.54 MPa, upon exposure to 1000 °C. The difference between the highest and the lowest strength was 51.31%. 

The strength of the DFA geopolymer increased as the temperature increased, attaining a peak strength of 74.48 MPa at 400 °C. At this stage, dolomite, which contains a high Ca content, promoted the optimum reaction to the geopolymerisation process. This is proven by the microstructure of composites, whereby all the raw materials had already reacted in the microstructure analysis earlier. The geopolymer matrix that had already undergone an optimum reaction was indicated by all the materials that lost their original shape. The strength development of the geopolymer using material that contained a high Ca composition was slow. The strength development in geopolymer could be accelerated by increasing the aging time, or when subjected to temperature exposure, or both. Thus, further introduction to heat (by increasing the temperature) up to a certain temperature accelerated the strength development of the geopolymer that contained high Ca materials. This further explains the increasing compressive strength after exposure to 400 °C.

The increase of 23% strength at 400 °C is attributed to the promotion of polycondensation between chain-like geopolymer gels. This can be attributed to the stiffening of gel and an increment in surface forces between the gel particles due to the release of adsorbed moisture or due to the promotion of polycondensation between chain-like geopolymer gels [43]. This is closely linked with the dehydration of free or weakly bound water content entrapped in the geopolymer during mixing and curing processes. Dehydration and oxidation often act, starting from 500 °C for OPC and other geopolymer materials. In some cases, the dehydration of the geopolymer matrix can lead to a significant reduction in strength, and some phase transformations occur after being exposed to temperatures ranging between 450 and 850 °C [6]. Regarding the geopolymer that contains dolomite with slow reactivity, the action occurred as early as 400 °C. At this stage, the DFA geopolymer was dry and did not contain entrapped water content, thus enhancing the strength of the composites. Heating the geopolymer composite to this temperature generated more high-density C-A-S-H with sodium aluminate silicate (N-A-S-H) phase. As the formation of C-A-S-H with N-A-S-H gel increased, the strength of the composites increased as well. More geopolymer phases were produced without the entrapped water content, resulting in the strength improvement of the geopolymer sample [44].

As the temperature increased from 600 to 1000 °C, the shrinkage and deterioration of the geopolymer sample started to occur for this composite. With increasing temperature, the pressure also increased in the geopolymer sample due to the expanding water vapour, which is also known as the dehydration and dehydroxylation of the binders. Differential thermal analysis of minerals revealed that dehydroxylation in the clay-like based occurred at varied temperatures, starting from ~500 or ~700 °C, or between 500 and 700 °C [30,45]. This pressure leads to shrinkage and cracking, thus decreasing the strength of the sample [37]. This is also proven by the change in peaks at 970 and 790 cm^−1^ in functional group analysis of the composite under 1000 °C from the control DFA composite. The strength deterioration from 600 to 1000 °C is attributable to the Ca(OH)_2_ decomposition that occurs, starting at 600 °C. Basically, Ca(OH)_2_ was generated from the decomposition of the C-A-S-H phase that contained Ca element due to exposure to elevated temperature [46]. The decomposition of C-A-S-H gels that took place in the DFA composite was due to the introduction of CaO. The CaO, which was derived from dolomite, may also cause severe strength reduction at high temperatures. Although the strength of DFA composites decreased after exposure to 1000 °C, the strength was still higher and better when compared to OPC-based composites.

## 4. Conclusions

The dolomite has the potential to be used as a raw material for geopolymer. However, it produces a low reactivity in the geopolymerisation process, which leads to slow strength development. Applying heat or temperature exposure to the DFA geopolymer composites appears to be successful in increasing the reactivity of the geopolymerisation process, thus helping to improve strength development. Surprisingly, the composite strength increased after being exposed to 400 °C. This outcome is considered unique, mainly because OPC, or a geopolymer using other materials, would start to decrease or retain their original strength. Based on the elemental distribution analysis, the elements in the geopolymer composites were distributed well, signifying a homogenous composite sample.

## Figures and Tables

**Table 1:** Chemical composition of dolomite.

Chemical Compound	Composition (wt %)
CaO	80.21
Al_2_O_3_	1.52
SiO_2_	2.50
Fe_2_O_3_	0.15
MgO	15.50
CuO	0.07
MnO	0.02

**Table 2:** Chemical composition of fly ash.

Chemical Compound	Composition (wt %)
SiO_2_	55.3
Al_2_O_3_	25.8
Fe_2_O_3_	5.5
CaO	2.9
MgO	0.8
SO_3_	0.3
